# Lanadelumab in a kidney transplant patient with hereditary angioedema due to C1-inhibitor deficiency and high cardiovascular risk - a case report

**DOI:** 10.3389/fimmu.2024.1472390

**Published:** 2024-09-27

**Authors:** Antonio Gidaro, Leyla La Cava, Mattia Donadoni, Valentina Popescu Janu, Chiara Cogliati, Antonio Luca Brucato, Andrea Zanichelli, Mauro Cancian, Emanuele Bizzi

**Affiliations:** ^1^ Department of Biomedical and Clinical Sciences “Luigi Sacco,” University of Milan, Luigi Sacco Hospital, Milan, Italy; ^2^ Internal Medicine Department, Fatebenefratelli Hospital, Milan, Italy; ^3^ Operative Unit of Medicine, Angioedema Center, IRCCS Policlinico San Donato, Milan, Italy; ^4^ Department of Biomedical Sciences for Health, University of Milan, Milan, Italy; ^5^ Department of Systems Medicine, University Hospital of Padua, Padua, Italy

**Keywords:** hereditary angioedema due to C1-inhibitor deficiency (HAE-C1INH), kidney failure, kidney transplantation, androgens, lanadelumab

## Abstract

**Introduction:**

Cardiovascular pathologies represent the first cause of death in uremic patients and are among the leading causes of mortality in patients with hereditary angioedema due to C1-inhibitor deficiency (HAE-C1INH). Before 2020, the most common treatment for long-term prophylaxis in HAE-C1INH patients in Italy was attenuated androgen, which may increase cardiovascular risk by multiple mechanisms.

**Case description:**

We present a case report of a 56-year-old patient with HAE-C1INH type I affected by IgA nephropathy with severe kidney impairment. The patient experienced a first kidney transplant and, after late rejection, underwent a second kidney transplant. Further comorbidities included obesity, hypertensive cardiomyopathy, HCV liver disease, and dyslipidemia. His prophylactic therapy to prevent angioedema attacks had consisted of attenuated androgens for about 40 years. Since 2020, new modern targeted therapy for LTP, particularly lanadelumab, has shown promising results. The majority of patients with attenuated androgens have been successfully switched to lanadelumab, including our patient. Since introducing lanadelumab (300 mg subcutaneously every two weeks; after a six-month attack-free period, the dosing interval of lanadelumab was extended to four weeks), the patient has not experienced any acute HAE attack and did not report any adverse events. Moreover, we observed decreased total cholesterol, C-LDL, and body mass index, reducing the Matsushita et al. score for ten years of cardiovascular risk from 13.2% to 9.3%.

**Conclusion:**

lanadelumab is effective and safe in preventing hereditary angioedema attacks, as well as in reducing cardiovascular risk in an immunosuppressed patient with significant comorbidities. The successful outcomes of this case highlight the potential of lanadelumab as a promising prophylactic therapy.

## Introduction

C1-inhibitor (C1INH) deficiency due to a mutation in the *SERPING1* gene is the most common cause of hereditary angioedema (HAE) (1, 2). Hereditary angioedema due to C1-inhibitor deficiency (HAE-C1INH) occurs worldwide, with an estimated prevalence in Italy of 1:64,000 (3). Clinical presentation usually consists of self-limiting, subcutaneous edema of the extremities, face, or genitals without wheals. Gastrointestinal and upper airway mucosa may also be involved with symptoms mimicking an acute abdomen or with the risk of asphyxiation, respectively (4). The mortality rate of HAE-C1INH is affected by the diagnostic delay and the availability of on-demand and prophylaxis treatment. However, in a retrospective study, asphyxiation in Italian HAE-C1INH families was less common than previously reported, with malignancy and cardiovascular disease representing the leading cause of death (5). Until 2020, when the kallikrein antagonist lanadelumab became available, the most common treatment for HAE-C1INH long-term prophylaxis (LTP) in Italy was danazol and stanazolol, two synthetic attenuated androgens (AA), which can predispose to cardiovascular and metabolic complaints (1). In January 2023, subcutaneous plasma-derived C1INH also arrived on the market and berotralstat in March 2024.

Zanichelli et al. recently found a greater incidence of hypertension (22.8% vs. 10.8%; OR 2.02), hypercholesterolemia (19.5% vs. 5.3%; OR 3.97), and diabetes mellitus (5% vs. 1.4%; OR 3.21) in HAE-C1INH patients on AA as LTP from a large cohort of the Italian network for Hereditary and Acquired Angioedema (ITACA). Renal failure was reported as a comorbidity in 3.8% of cases, with AA as a risk factor (OR 1.58 from 0.59 to 4.24) (6). For this reason, especially in uremic patients in whom cardiovascular diseases represent the first cause of death (7), it is essential to discontinue AA treatment. Here, we report a case of an immunosuppressed kidney transplant patient with HAE-C1INH who discontinued AA prophylaxis and switched to Lanadelumab LTP.

## Case description

**Figure 1:**
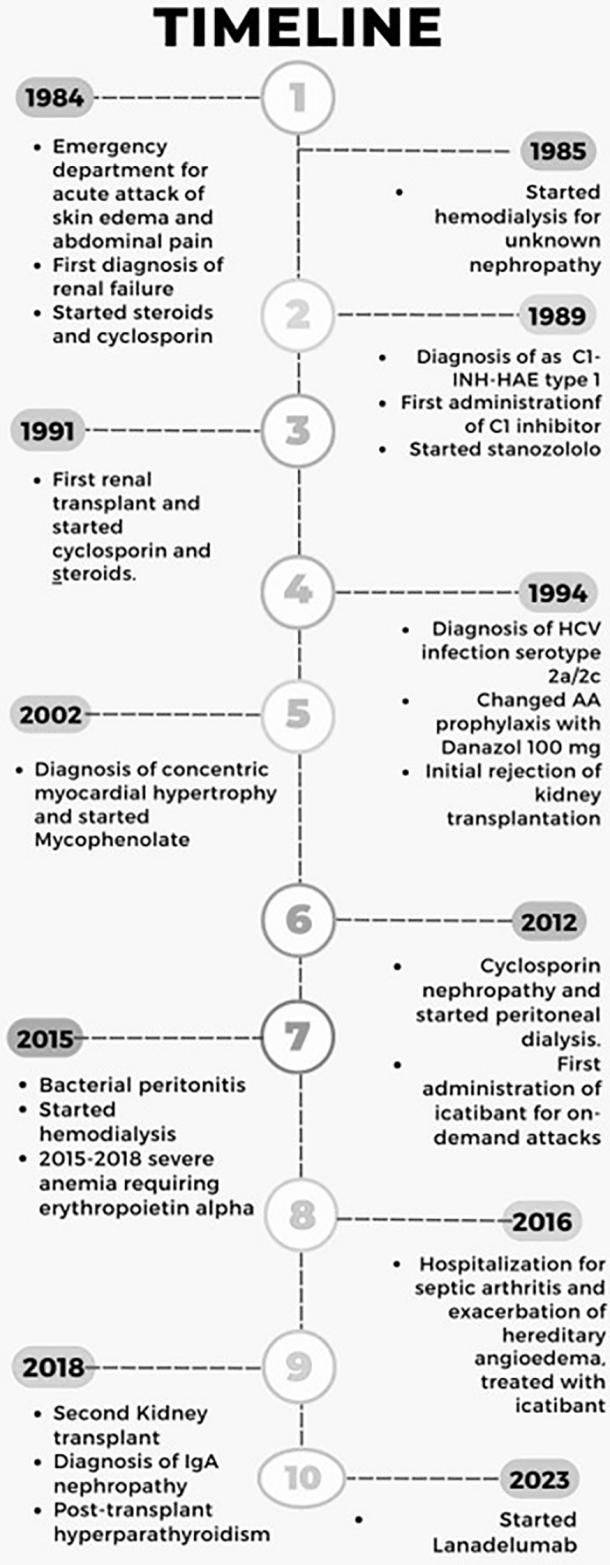
Timeline of the patient journey from the first C1INH-HAE episode in 1984 until today. Written informed consent was obtained from the individual to publish any potentially identifiable images or data in this article.

A male patient, 57 years old, presented in 1984, at the age of 17, to the Emergency Room for his first episode of angioedema, affecting one arm (**Figure 1**). Blood analysis showed evidence of previously unknown acute renal failure, and soon after, he started immunosuppressive therapy with steroids and cyclosporin. Unfortunately, disease progression led the patient to hemodialysis in the next year, and at that time, no clear definition of nephropathy was provided. In the following five years, he continued to experience a mean of two attacks per month of angioedema with localization on the face or limbs that were treated with standard therapy (corticosteroids and antihistamines) without regression of symptoms. Finally, on January 1989 he was diagnosed as HAE-C1INH type I (C1-INH antigen: 0.02 g/L; normal range: 0.21-0.38 g/L; Functional C1INH activity:10%; normal range: 70-130%; C4: 0.03g/L; normal range 0. 1-0.4 g/L). C1q plasma level was normal (0.23 g/L; referral range: 0.1-0.25 g/L), a common finding in hereditary angioedema. Family screening tested negative and classified the patient as a *de novo* mutation. Unfortunately, his children would have been later affected by angioedema, thus confirming the diagnosis of HAE-C1INH type I. The patient was treated with on-demand plasma-derived C1INH concentrate administered intravenously in case of an acute attack with rapid regression of angioedema symptoms. However, the frequency of episodes prompted us to prescribe LTP with stanozolol 4 mg QD in March 1989. Before AA treatment, he discovered an HCV infection serotype 2a/2c. This liver disease was considered, but due to the high number of angioedema attacks, AA therapy was started with strict control of liver functional exams and ultrasound evaluation every six months. The attenuated androgen was well tolerated and effective in preventing recurrences of angioedema, reduced to only one attack per year. After one year of treatment, the dose of stanozolol was reduced to 1 mg with sustained control of attacks. In October 1991, the patient underwent a kidney transplant, and therapy with steroids and cyclosporine was reintroduced. Shortly after, he developed arterial hypertension treated with beta-blockers. In 1994, he changed AA prophylaxis, switching to Danazol at 100 mg due to the lack of stanazolol on the market. This change didn’t affect the efficacy of AA prophylaxis. Due to the development of concentric myocardial hypertrophy, a steroid-sparing immunosuppressive treatment with mycophenolate was started in 2002 when the patient was 35 years old. Doxazosin and amlodipine were added as antihypertensive drugs, and the clinical situation remained stable until 2012, when he developed cyclosporin nephropathy, leading to transplant failure with the need for peritoneal dialysis. This procedure, probably together with psychological stress, resulted in a recrudescence of HAE attacks, and the dose of Danazol was increased to 100 mg BID – despite comorbidities - to obtain disease control. After bacterial peritonitis in 2015, hemodialysis through a central venous catheter three times per week was recommended. Finally, after the onset of severe anemia requiring alpha erythropoietin administration, two catheter-related systemic infections, and the exclusion of active HCV infection, he performed a second kidney transplant in 2018. Histopathology from the removed kidney was consistent with IgA nephropathy. Since then, immunosuppressive treatment with mycophenolate 1000 mg, tacrolimus 1 mg, and prednisone 5mg QD is still ongoing together with calcium-mimetic therapy (Cinacalcet 30 mg) for persistent, post-transplant hyperparathyroidism.

In June 2023, when the patient was 56 years old, we calculated the cardiovascular risk during a follow-up visit, according to the Matsushita et al. score incorporating kidney disease measures into cardiovascular risk prediction (8), resulting in 13.2% at ten years (9). Furthermore, the patient reported weight gain (BMI 31.2 kg/m2) as an adverse event of AA. Therefore, although HAE-C1INH symptoms were well controlled with Danazol 100 mg BID effect. A novel prophylactic treatment for HAE-C1INH was proposed to reduce cardiovascular risk. After careful evaluation of the patient’s need and extensive discussion with him, he gradually reduced the posology of danazol 50 mg every two weeks until discontinuation in 8 weeks. Then, he started lanadelumab 300 mg SC every two weeks. After a six-month attack-free period, the dosing interval of lanadelumab was extended to four weeks. The patient is still asymptomatic for angioedema; he lost 4 kg in weight, and the cardiovascular risk is now 9.3% at ten years, thanks to improved lipid metabolism (**Table 1**).

**Table 1:** Modified cardiovascular risk prediction score by Matsushita et al. This calculator incorporates the kidney disease measures of estimated glomerular filtration rate and urine albumin to creatinine ratio to enhance the prediction for Cardiovascular Risk.

Before Lanadelumab 13.2%	Values
- eGFR (ml/min/1.73 m^2^) - Urine albumin to creatinine ratio (mg/g) - Age (years) - Gender - Race - Systolic blood pressure - Antihypertensive medications - HDL cholesterol (mg/dl) - Total cholesterol (mg/dl) - Diabetes - Current smoker	64 57 Male Caucasian Yes 41 220 No No
**Total score**	**13.2%**
After six months of Lanadelumab treatment	
- eGFR (ml/min/1.73 m^2^) - Urine albumin to creatinine ratio (mg/g) - Age (years) - Gender - Race - Systolic blood pressure - Antihypertensive medications - HDL cholesterol (mg/dl) - Total cholesterol - Diabetes - Current smoker	80 57 Male Caucasian Yes 48 180 No No
**Total score**	**9.2%**

## Discussion

According to the latest WAO-EAACI 2022 guidelines for hereditary angioedema, four treatments are indicated for LTP in patients with HAE-C1INH: berotralstat, lanadelumab, and subcutaneous or intravenous plasma-derived C1INH concentrates. All these drugs show a safe cardiovascular profile. At the same time, attenuated androgens are now considered a second-choice treatment to be used only if specific medications are not available, as they may induce several side effects resulting in increased cardiovascular risk (1, 5, 6).

While there are no consensus guidelines on how best to stop AAs, several real-world strategies have been described for transitioning from AAs to first-line LTP. These strategies are based on tapering, overlapping treatments, or an immediate switch (10). The latter approach has raised concerns regarding an increase in HAE attacks and other adverse events (10). It is recommended to avoid abrupt withdrawal of AAs when transitioning to novel prophylaxis to minimize side effects from AA withdrawal. In our case, we decided to taper the androgen dose with reliable access to on-demand medication to treat HAE symptoms to reduce the anxiety related to androgen discontinuation in a patient with thirty-four years of AA treatment. This approach can be individualized based on the duration of androgen therapy, adverse effects experienced, transition plans to another treatment and patient anxiety related to androgen discontinuation.

Our patient didn’t experience increased HAE attacks during the eight weeks of AA tapering. However, we decided to start the new LTP to minimize the patient’s burden of disease, who experienced a lot of medical procedures and complications in his whole life.

The plasma kallikrein inhibitor Berotralstat might theoretically represent, given the oral route of administration, the most practical alternative to attenuated androgens. However, adverse effects include urinary tract infection and diarrhea (APEX study, 9.9% and 6.2% of patients, respectively) (11) that could lead to a worsening of renal failure, especially in kidney transplanted patients. Moreover, Berotralstat presents some relevant drug-to-drug interference (**Table 2**), which suggests not considering this drug, available in Italy only since March 2024, as the best option to replace attenuated androgens in patients such as the current case report.

**Table 2:** Notable drug interactions between berotralstat and patient’s medications.

Concomitant medication	Possible effect	Recommendation during berotralstat treatment
**Tacrolimus**	- berotralstat will increase the level or effect of tacrolimus by P-glycoprotein (MDR1) efflux transporter - berotralstat will increase the level or effect of tacrolimus by affecting hepatic/intestinal enzyme CYP3A4 metabolism	Use Caution/Monitor
**Prednisone**	- berotralstat can increase the drug level of prednisone. Coadministration with inhibitors of CYP450 3A4 may increase the plasma concentrations and pharmacologic effects of corticosteroids, primarily metabolized by the isoenzyme.	The possibility of increased corticosteroid effects should be considered during coadministration with potent and moderate CYP450 3A4 inhibitors.

Subcutaneous and intravenous plasma-derived C1-INH concentrates represent another approach for LTP in C1INH deficiency. This LTP aims to replace the lacking protein without any potential drug interactions. However, local adverse events may occur (site of injection-related pain, hematoma, bleeding, induration, bruising) mostly in older HAE-C1INH patients (12), and there is a relevant burden of treatment mainly related to the interval of administration, which is every 3-4 days for both the available formulations. Moreover, our patient used to travel to foreign countries for at least one month every year; thus, carrying a high number of vials for LTP results with him during his travel is unfeasible (12).

Lanadelumab, a human monoclonal antibody targeting plasma kallikrein, effectively prevents angioedema attacks in patients with HAE-C1INH. Minor injection site reactions, such as pain, redness, and bruising, are present in 42.9% of patients but do not represent a relevant complaint nor a cause of discontinuation in real life (13). Subcutaneous Lanadelumab must be injected every 14 days at 300 mg (1 vial sc) for the first six months. The interval can be extended to every four weeks if adequate control is achieved.

No dedicated studies have been conducted to evaluate the pharmacokinetics of Lanadelumab in patients with renal failure, but based on pharmacokinetic analysis, renal impairment (estimated GFR: 60 to 89 mL/min/1.73m2, [mild, N=98] and 30 to 59 mL/min/1.73m2, [moderate, N=9]) does not affect the clearance or volume of distribution of Lanadelumab. Our patient’s GFR was over 30mL/min/1.73m2, thus satisfying this inclusion criterion.

Concomitant medications such as analgesic, antibacterial, antihistamine, anti-inflammatory, and anti-rheumatic medications did not affect the clearance and volume of distribution of Lanadelumab during the Randomized control trial. Moreover, Cytochrome P450 enzymes, efflux pumps, and protein binding mechanisms are not involved in the clearance of human monoclonal antibodies, thus limiting potential interactions (14) in patients on multiple therapies, as in our case.

The concern we had when the patient was switched from androgens to lanadelumab was the potential effect of adding lanadelumab to the ongoing immunosuppressive treatment, as a higher rate of upper respiratory infection had been reported compared with placebo (44% vs. 32%) in the loading dose period of 300 mg every two weeks (13).

During the 13-month follow-up period, our patient showed no evidence of infections or adverse side effects.

## Conclusion

Lanadelumab was effective and safe as LTP for HAE-C1INH in an immunosuppressed patient, kidney transplant recipient, and with multiple comorbidities.

Replacing attenuated androgens with lanadelumab has also improved metabolic control and reduced cardiovascular risk.

## Data Availability

The raw data supporting the conclusions of this article will be made available by the authors, without undue reservation.
